# mRNA Vaccine for Alzheimer’s Disease: Pilot Study

**DOI:** 10.3390/vaccines12060659

**Published:** 2024-06-14

**Authors:** Armine Hovakimyan, Garri Chilingaryan, Olga King, Joia Kai Capocchi, Jean Paul Chadarevian, Hayk Davtyan, Roman Kniazev, Michael G. Agadjanyan, Anahit Ghochikyan

**Affiliations:** 1 The Institute for Molecular Medicine, Huntington Beach, CA 92647, USA; ahov@immed.org (A.H.); garri@immed.org (G.C.); osvystun@uci.edu (O.K.); rkniazev@immed.org (R.K.); 2 The Institute for Memory Impairments and Neurological Disorders, The University of California, Irvine, CA 92697, USA; jcapocch@uci.edu (J.K.C.);; 3 Sue and Bill Gross Stem Cell Research Center, University of California, Irvine, CA 92697, USA

**Keywords:** mRNA vaccine, DNA vaccine, Alzheimer’s, humoral immune responses, immunogenicity, pathological amyloid-β, mice, non-human primates

## Abstract

The escalating global healthcare challenge posed by Alzheimer’s Disease (AD) and compounded by the lack of effective treatments emphasizes the urgent need for innovative approaches to combat this devastating disease. Currently, passive and active immunotherapies remain the most promising strategy for AD. FDA-approved lecanemab significantly reduces Aβ aggregates from the brains of early AD patients administered biweekly with this humanized monoclonal antibody. Although the clinical benefits noted in these trials have been modest, researchers have emphasized the importance of preventive immunotherapy. Importantly, data from immunotherapy studies have shown that antibody concentrations in the periphery of vaccinated people should be sufficient for targeting Aβ in the CNS. To generate relatively high concentrations of antibodies in vaccinated people at risk of AD, we generated a universal vaccine platform, MultiTEP, and, based on it, developed a DNA vaccine, AV-1959D, targeting pathological Aβ, completed IND enabling studies, and initiated a Phase I clinical trial with early AD volunteers. Our current pilot study combined our advanced MultiTEP technology with a novel mRNA approach to develop an mRNA vaccine encapsulated in lipid-based nanoparticles (LNPs), AV-1959LR. Here, we report our initial findings on the immunogenicity of 1959LR in mice and non-human primates, comparing it with the immunogenicity of its DNA counterpart, AV-1959D.

## 1. Introduction

Alzheimer’s Disease (AD) is a leading cause of dementia, with over 55 million individuals impacted worldwide and representing up to 70% of all dementia cases. Its profound effects stretch far beyond patients themselves, deeply affecting families and placing a challenging burden on the broader social and economic structures. From 2000 to 2019, while mortality rates for stroke, heart disease, and HIV declined in the United States, reported deaths from AD surged by over 145%, leading to officially recorded 121,499 deaths in 2019 alone, making AD the sixth leading cause of death in the country [1].

The escalating global healthcare challenge posed by AD and compounded by the lack of effective treatments emphasizes the urgent need for innovative approaches to combat this devastating disease. Among the diverse therapeutic interventions explored, passive immunotherapies with monoclonal antibodies (mAbs) and vaccines stand out as the most reliable and promising strategies. Recent data with FDA-approved mAbs (aducanumab and lecanemab) specific to the N-terminal region of amyloid-β (Aβ) that preferentially target Aβ fibrils and protofibrils, respectively, have provided the first solid evidence that clearance of Aβ aggregates from the brains of people with early AD can slow down the progression of AD [2,3,4,5]. Although both mAbs received FDA approval, only lecanemab (Leqembi^®^) is currently used for biweekly intravenous (IV) treatment (10 mg/kg/injection) of people with early AD. Even though the clinical benefits observed with Leqembi^®^ treatment have been modest, they demonstrated the significance of early intervention in the pathogenic cascade of AD.

These clinical data support our long-time hypothesis based on our nonclinical studies suggesting that antibodies can inhibit oligomerization of pathological Aβ and delay the onset of dementia when treatment is initiated in cognitively unimpaired individuals at risk of AD. However, employing mAbs as a preventive measure is impractical due to their intricate nature, substantial cost, and the necessity for frequent high-dose intravenous administrations. Additionally, there is a significant risk of amyloid-related imaging abnormalities, including cerebral microhemorrhages (ARIA-H) and edema (ARIA-E). For example, the approval of lecanemab does not extend to the treatment of pre-symptomatic individuals at risk of AD due to the observed occurrences of symptomatic brain edema (ARIA-E) and intracranial bleeding (ARIA-H) in treated individuals. Conversely, over 17 years ago, we decided to develop an immunogenic and safe vaccine for the prophylactic treatment of cognitively unimpaired individuals over 50 years old at risk of AD. To generate therapeutically potent antibodies in asymptomatic, vaccinated people, we developed a novel vaccine platform, MultiTEP [6,7,8,9,10,11]. This innovative vaccine platform consists of a string of promiscuous foreign T helper (Th) cell epitopes derived from pathogens and a synthetic Th epitope called PADRE [12,13]. Unlike other carriers used in AD vaccine trials (e.g., KLH, Qβ VLP, CRM197, etc.), each Th epitope within the MultiTEP has been meticulously selected for its proven immunological activity in humans [14,15,16,17]. Importantly, the Th epitope sequences are chosen from tetanus, hepatitis B (HBV), and influenza, pathogens that individuals are commonly exposed to or vaccinated against during their lifetime. 

As a result, vaccinated subjects possess memory Th cells that can mount rapid and robust responses to the MultiTEP platform. The design of this platform is specifically tailored to older people with immunosenescence [18,19]. Its objectives include (i) overcoming self-tolerance; (ii) activating both naïve and pre-existing memory Th cells generated in response to conventional vaccines or infections with indicated pathogens during one’s lifespan; (iii) avoiding the generation of potentially harmful autoreactive T cells specific to molecules involved in AD pathology; and (iv) providing broad coverage of high *MHC class II* gene polymorphisms in humans. 

Using the universal MultiTEP platform technology, we have developed DNA-based vaccines targeting molecules involved in the pathogenesis of various neurodegenerative disorders, and we have extensively investigated the immunogenicity and efficacy of these vaccines across relevant disease mouse models, healthy rabbits, and aged non-human primates (NHPs). Of note, bearing in mind that DNA vaccines induce weaker immune responses in NHPs and humans compared with mice [20], we delivered DNA intramuscularly or intradermally by using a gene gun, electroporation, and high-pressure jet systems [6,7,8,21]. Furthermore, we initiated a Phase I trial with one of these MultiTEP-based vaccines targeting the B cell epitope of amyloid, Aβ_1–11_, AV-1959D. In this FDA-cleared study, the participants with early AD were immunized intradermally with the AV-1959D vaccine using a needle-free Pharmajet device, Tropis (NCT05642429, Pharmajet, Golden, CO, USA). 

In this pilot study, we synthesized an mRNA based on the AV-1959 antigen composed of MultiTEP and three copies of a peptide spanning aa 1–11 of amyloid (Aβ_42_). It is worth noting that because this is our initial attempt to test the mRNA for developing an AD vaccine, we did not specifically optimize the mRNA sequence for the expression of AV-1959. Following encapsulating mRNA into lipid nanoparticles (LNPs), we developed a prototype mRNA vaccine called AV-1959LR. This vaccine shows promise as an alternative to the DNA vaccine AV-1959D due to its easy delivery method by needle, more robust immunogenicity, and lower manufacturing costs. Below, we showed the immunogenicity of the AV-1959LR vaccine and compared it with the humoral immune responses of AV-1959D in mice and monkeys. 

## 2. Materials and Methods

### 2.1. Animals

Seven-week-old female C57BL/6 mice (H-2b haplotype) were obtained from the Jackson Laboratory in Sacramento, CA, USA. These mice were kept in a controlled environment with regulated temperature and light cycles, following the guidelines set by the National Institutes of Health and an approved protocol from the Institutional Animal Care and Use Committee (IACUC) at the University of California, Irvine, CA, USA. Furthermore, 10-year-old adult female cynomolgus monkeys (*Macaca fascicularis*) from the Alpha Genesis, Inc. primate colony in Yemassee, SC, USA were used in this study. The monkeys were housed according to established standards and monitored daily for any signs of clinical abnormalities or distress, including changes in food intake, activity levels, appearance, and stool consistency, as previously documented [11].

### 2.2. Vaccine Preparation

**Figure 1:**
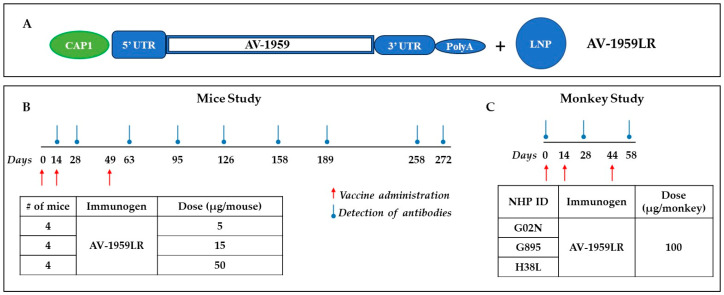
Pilot study design for testing messenger RNA (mRNA) vaccine for Alzheimer’s targeting N-terminus of pathological amyloid, Aβ. Schematic representation of AV-1959LR based on Vernal proprietary vector, which is not specifically optimized for AV-1959 vaccine (**A**). Design of experimental protocol for mice (**B**) and non-human primates (**C**) vaccinated with AV-1959LR. The red arrow shows the days of vaccine administration, and the blue circled arrow shows when blood was drawn to detect Aβ-specific antibodies in sera.

*mRNA synthesis:* AV-1959LR was prepared at Vernal Biosciences. A DNA sequence comprising proprietary 5′ UTR and 3′ UTR, the poly-A region, and the AV-1959 gene was generated using splicing overlap extension of oligonucleotides and cloned into the plasmid DNA (pVRN). pVRN-AV1959 was then linearized with a type IIs restriction enzyme at the end of the polyA region, purified, and incubated with the DNA-dependent RNA polymerase, natural and modified nucleotides (N1-methyl-pseudouridine-Triphosphate), and an optimized buffer system in the in vitro transcription (IVT) reaction. The full-length mRNA was affinity-purified following pDNA template elimination using DNAse-I and capped using the Vaccinia capping enzyme system. The capped and nucleoside-modified mRNA were subjected to quality control prior to formulation (Figure 1A). 

*LNP Formulation:* LNP formulation involves mixing lipids and mRNA, which spontaneously nanoprecipitates into LNP –mRNA. The mixture was diluted to reduce the percent of ethanol (used to dissolve the lipids) and subjected to tangential flow filtration (TFF) for buffer exchange into a freezer-stable, clinically injectable buffer. LNP composition is 50% ionizable lipid, 38.5% cholesterol, 10% phospholipid, and 1.5% lipid-PEG.

### 2.3. Immunization of Mice

Three groups of mice were immunized with 5 μg/mouse (n = 4), 15 μg/mouse (n = 4), and 50 μg/mouse (n = 4) AV-1959LR on days 0, 14, and 49 via intramuscular injection (Figure 1B). Blood samples were collected on days 14, 28, 63, 95, 126, 158, 189, 258, and 272. The serum was then separated and used to analyze humoral immune responses and to characterize antibodies.

### 2.4. Vaccine Administration to Non-Human Primates

Monkeys were injected with 100 μg of AV-1959LR vaccine on days 1, 14, and 44 intramuscularly (Figure 1C). Blood was collected on days 1 (baseline), 28, and 58. The serum was then separated and used to analyze humoral immune responses and characterize antibodies.

### 2.5. Detection of Aβ-Specific Antibodies 

The concentrations and endpoint titers of anti-Aβ antibodies in mice and monkey sera were determined through enzyme-linked immunoassay (ELISA), as described previously [8,10,21]. Briefly, plates were coated with Aβ42 peptide (GenScript, Piscataway, NJ, USA). For mouse sera, HRP-conjugated goat anti-mouse IgG (1:2500; Jackson ImmunoResearch, West Grove, PA, USA) was used as the secondary antibody, whereas for monkey sera, HRP-conjugated goat anti-monkey IgG (1:20,000; Invitrogen, Waltham, MA, USA) was utilized. The reaction was developed with 3,3′,5,5′-tetramethylbenzidine (TMB) substrate solution and stopped with 2 N H_2_SO_4_. Optical density readings were taken at 450 nm using a FilterMax F5. Antibody concentrations in mouse sera were calculated using a calibration curve generated with the 6E10 monoclonal antibody (Biolegend, San Diego, CA, USA). Endpoint titers in monkey sera were determined as the reciprocals of the highest serum dilutions, yielding an optical density reading three times higher than the cutoff, which was established based on the titer of pre-immune sera at the same dilution.

The isotypes of monkey anti-Aβ antibodies were evaluated in serum diluted 1:2000 using horseradish peroxidase (HRP)-conjugated anti-monkey IgG (Fitzgerald Industries, Inc., Acton, MA, USA) and anti-monkey IgM (Alpha Diagnostic Intel, Inc., San Antonio, TX, USA) secondary antibodies at dilutions of 1:50,000 and 1:2000, respectively. 

### 2.6. Epitope Mapping of Aβ-Specific Antibodies

Fine epitope mapping of anti-Aβ antibodies was conducted using an “alanine scanning” approach with competition ELISA, as described in [10]. Briefly, 11 peptides were synthesized spanning the Aβ1–11 sequence, with each peptide having one alanine substitution at a different position. Serial dilutions of reference non-mutated (Aβ1–11) or mutated test peptides (at final concentrations of 0.02 μM, 0.1 μM, 0.5 μM, 2.5 μM, 5 μM, and 12.5 μM) were incubated with diluted immune sera (at 1:170,000 for mouse sera and 1:900 for monkey sera) for 1.5 h at 37 °C. After incubation, the binding of the antibody/peptide mixture to Aβ_42_ was detected using standard ELISA. The percentage of binding of sera blocked with peptides to Aβ_42_ was calculated, with the binding of sera without competing peptides to Aβ_42_ considered 100%. The half-maximal inhibitory concentration (IC50) for each peptide was calculated.

### 2.7. Detection of Aβ Plaques in Human Brain Tissues through Immunofluorescence and Confocal Microscopy

Sera from mice and monkeys immunized with AV-1959LR and AV-1959D were examined for their capacity to bind to human Aβ plaques. First, 30 μm brain sections were utilized from formalin-fixed cortical tissues of a severe AD case and a non-AD control case, sourced from the Brain Bank and Tissue Repository at MIND, UC Irvine. The brain sections underwent PBS rinsing and a 1 h blocking step in PBS containing 0.05% Triton-X and 10% donkey serum. Samples were stained with AmyloGlo™ RTD Amyloid Plaque Stain Reagent (Biosensis, Thebarton, SA, Australia) for 15 min, followed by three washes in PBS. Subsequently, they were incubated overnight at 4 °C with immune sera from mice or monkeys diluted to 1:100. The following day, the sections were washed three times in PBS and incubated with species-specific Alexa-Fluor-conjugated secondary antibodies at room temperature for 1 h. After three additional PBS washes, the sections were mounted on slides and cover-slipped with Fluoro-mount-G. Immunofluorescent staining was conducted on equivalent brain sections and imaged at 20× magnification with a 3.72 zoom on an Olympus FX1200 confocal microscope (Olympus, Tokyo, Japan). β-amyloid plaques were visualized using Z-stack maximum-projection images taken at 1.25 μm intervals through the entire depth of the section.

### 2.8. Statistical Analysis 

Statistical parameters (mean ± SEM, significant differences, IC50, etc.) were calculated using the Prism 10 software (GraphPad Software, Inc., La Jolla, CA, USA). Statistically significant differences were examined using a one-way ANOVA multiple comparison test. We used standard designations of *p* values throughout the figures (ns = not significant or *p* ≥ 0.05; * *p* < 0.05; ** *p* < 0.01; *** *p* < 0.001; **** *p* < 0.0001).

## 3. Results

### 3.1. AV-1959LR, an mRNA-Based Vaccine: The Counterpart of the AV-1959D DNA Vaccine

In this pilot study, we developed the mRNA counterpart of our MultiTEP-based AV-1959D DNA vaccine using Vernal’s proprietary vector for mRNA synthesis (Figure 1A). Specifically, the mRNA encodes the AV-1959 protein, which comprises three copies of the N-terminal region of human Aβ spanning amino acids 1–11, attached to an immunogenic vaccine platform, MultiTEP, consisting of twelve foreign promiscuous T helper (Th) cell epitopes, including one synthetic peptide (PADRE), eight epitopes from Tetanus Toxin (TT) (P2, P21, P23, P30, P32, P7, P17, and P28), two epitopes from HBV surface antigen (HBsAg, aa 19–33) and the nucleocapsid (HBVnc, aa 50–69), respectively, and one epitope from influenza virus matrix protein (MT, aa 17–31). The mRNA synthesis involved the incorporation of modified N1-methylPseudoUridine and capping (CAP1) at the 5′ end. The AV-1959 mRNA was then encapsulated in ALC-0315 LNPs, resulting in the final vaccine formulation, which was designated AV-1959LR.

### 3.2. Immunogenicity and Dose–Response to AV-1959LR Vaccine in Mice

**Figure 2:**
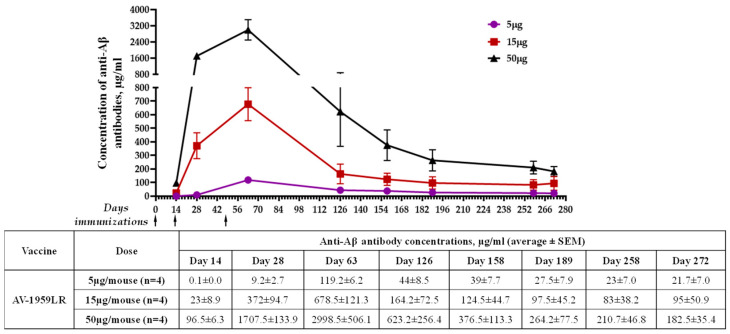
Humoral immune responses induced in C57BL/6 mice immunized with AV-1959LR. Dynamics of antibody production in mice immunized with 5 μg, 15 μg, and 50 μg doses of AV-1959LR. Concentrations of anti-Aβ antibodies were determined through ELISA, as described in the Materials and Methods, and calculated using a calibration curve generated with 6E10 mouse monoclonal antibody (mAb). Each point represents the mean value of antibody concentration (average ± SEM). The antibody concentrations in mice from the high-dose group on days 14, 28, and 63 were significantly higher compared to those in the medium- and low-dose groups (*p* < 0.05). The difference was not significant on day 126 (*p* ≥ 0.05), and, starting from day 158 to the day 272, a significant difference was observed only between the high- and low-dose groups (*p* < 0.05).

The immunogenicity of AV-1959LR was evaluated in C57BL6 mice immunized with different doses of vaccine. Schedules of immunization and vaccine doses are shown in Figure 1B. The assessment of humoral immune responses following each immunization demonstrated that AV-1959LR elicited antibody production in all vaccinated mice, exhibiting a pronounced dose-dependent effect (Figure 2A). Notably, mice from the high-dose group displayed significantly higher antibody concentrations than those from the medium-dose group (*p* < 0.001), which, in turn, exhibited significantly higher concentrations compared to the low-dose group (*p* < 0.01). Following the third immunization, the mean antibody concentration in the high-dose group was approximately 2998 μg/mL, whereas it was 678 μg/mL and 119 μg/mL for the medium- and low-dose groups, respectively. Remarkably, even mice from the low-dose group produced substantial antibody concentrations, which, as we have demonstrated previously, are sufficient for clearing amyloid plaques in the brains of vaccinated mice. Monitoring antibody responses over 10 months revealed a gradual decline in antibody titers across all groups. However, by day 272, antibodies were still detectable in all vaccinated mice, with significantly higher concentrations observed in the high-dose group compared with the low-dose group (*p* < 0.05). 

Thus, our pilot study demonstrated that a 50 µg/mouse dose of AV-1959LR led to exceptionally high levels of humoral immune response, making it a promising prototype vaccine for AD.

### 3.3. Epitope Mapping of Anti-Aβ Antibodies Generated by AV-1959LR in Mice

**Figure 3:**
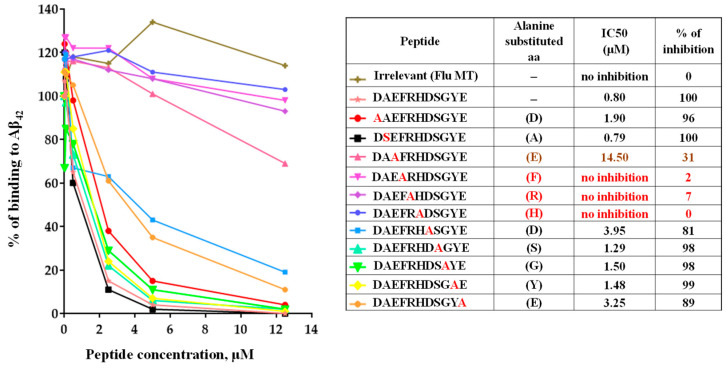
Epitope mapping of antibodies generated in mice immunized with AV-1959LR vaccine was performed using alanine scanning competition ELISA. Alanine substitution of each amino acid (alanine is marked in red) in the Aβ1–11 peptide demonstrated vaccine-induced production of anti-Aβ antibodies that strongly recognized the EFRH sequence of the Aβ peptide. IC50 and percent of inhibition of antibody binding to Aβ42 peptide with 12.5 μM of mutated peptides were calculated, considering the binding of sera without competing peptides to Aβ42 as 100%. The epitope amino acids are indicated by color, with the most essential amino acids for binding marked in red.

Previously, we demonstrated that mice immunized with AV-1959D generated antibodies primarily binding to the AEFRH epitope [8]. To confirm that the mRNA-based vaccine induces antibodies of the same specificity, we conducted fine epitope mapping for antibodies generated by AV-1959LR in mice. Using alanine scanning competition ELISA, we showed that substitutions of amino acids at positions 3–6 of the Aβ_42_ peptide to alanine affected the ability of antibody to bind to the Aβ_42_ peptide, mapping the epitope to EFRH, with FRH being the most crucial amino acids (Figure 3). In contrast, an irrelevant peptide showed no inhibition of binding, underscoring the specificity of the antibody response to the Aβ_42_ peptide. 

### 3.4. Binding of Anti-Aβ Antibodies to Amyloid Plaques

**Figure 4:**
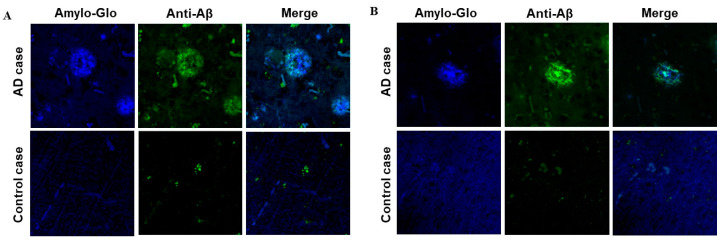
Antibodies generated by AV-1959LR (**A**) and AV-1959D (**B**) specifically recognize Aβ plaques in the brains of AD cases. However, AV-1959LR-induced antibodies primarily recognize plaque-associated halo, whereas AV-1959D-induced antibodies bind to both the halo and the dense core of the plaque. There was no binding observed in control non-AD brain sections.

We evaluated the ability of AV-1959LR-induced antibodies to recognize human senile plaques and compared them with antibodies induced by the AV-1959D DNA vaccine through immunofluorescent staining of brain sections from an AD case. As anticipated, sera from mice immunized with both AV-1959LR and AV-1959D bound to amyloid plaque pathology (Figure 4). The specific binding of sera (green) to amyloid plaques was demonstrated by co-labeling brain sections with amylo-Glo (RTD Amyloid Plaque Stain Reagent). An intriguing finding was that antibodies generated by the AV-1959LR vaccine predominantly bound to the halo of medium and small non-fibrillar Aβ entities surrounding the plaque. In contrast, antibodies generated by the AV-1959D vaccine bound to both the halo and the dense core formed by higher-order Aβ structures [22]. While this observation is noteworthy, it requires validation with more vaccinated animals and brain sections from various AD cases. 

### 3.5. Immunogenicity of AV-1959LR mRNA Vaccine in NHPs 

Further studies on the immunogenicity of AV-1959LR were conducted in NHPs (Macaca fascicularis, n = 3), which naturally exhibit tolerance to Aβ_42_ and possess diverse genetic profiles for MHC class II molecules, closely resembling the human MHC class II region and repertoire [23]. Consequently, NHPs are a more relevant choice for evaluating the immunogenicity of MultiTEP-based AD vaccines. The monkeys were immunized on days 0, 28, and 58, with 100 µg of AV-1959LR per administration. Antibody titers were measured 14 days after the second and third immunizations (Figure 1C). 

**Figure 5:**
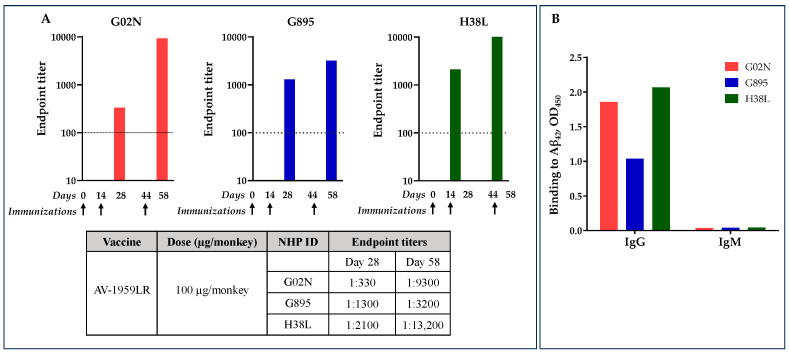
AV-1959LR-induced production of anti-Aβ antibodies of IgG isotype in Macaca fascicularis. Endpoint titers of antibodies were evaluated in individual NHPs at days 28 and 58 after 2nd and 3rd immunizations, respectively. (**A**). The dashed line represents the baseline at day 0. Arrows show days of immunization. AV-1959LR-induced antibodies of IgG isotypes indicate that the humoral immune responses were T-cell-dependent (**B**).

Antibody titers were relatively low after the second immunization but significantly boosted after the third immunization (Figure 5A). After the second immunization, G02N showed a lower response compared to the other two animals, which responded equally well. However, after the third immunization, the response in G02N was substantially elevated, whereas the boost in G895 was relatively modest. Monkey H38L was the highest responder, showing a significant boost in antibody titers after the third immunization.

As we expected from the previous studies [7], the isotype distribution of the elicited antibodies was predominantly of the IgG class, with negligible IgM response detected across all monkeys (Figure 3), indicating that the humoral immune responses were T-cell-dependent. 

It is crucial to highlight that despite the mRNA not being optimized, these compelling results are promising and strongly indicate the potential for achieving significant vaccine immunogenicity through mRNA optimization strategies, such as codon optimization and UTR scouting/selection.

### 3.6. Epitope Mapping of Anti-Aβ Antibodies Generated by AV-1959LR in Non-Human Primates

**Figure 6:**
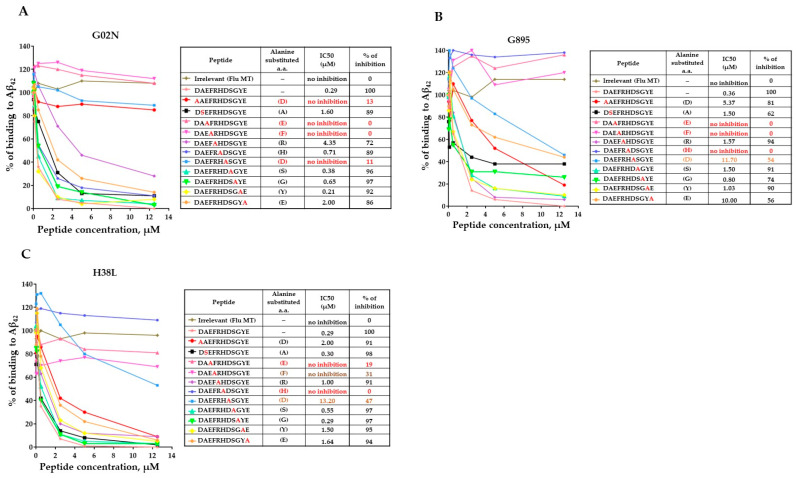
Epitope mapping of antibodies generated in NHPs immunized with AV-1959LR vaccine was performed using alanine scanning competition ELISA. In monkey G02N (**A**), generated antibodies strongly recognized the DAEFRHD epitope with the most essential amino acids D, E, F, and D. Anti-Aβ antibodies produced in monkeys G895 (**B**), and H38L (**C**) recognized the EFRHD epitope with the most essential amino acids E, F, and H. IC50 and percent of inhibition of antibody binding to Aβ42 peptide with 12.5 μM of mutated peptides were calculated, considering the binding of sera without competing peptides to Aβ42 as 100%. The epitope amino acids are indicated by color, with the most essential amino acids for binding marked in red.

Recently, the FDA approved monoclonal antibodies specific to the N-terminal region of amyloid for Alzheimer’s Disease treatment [24]. Our published studies demonstrated that monkeys vaccinated with AV-1959D generated antibodies recognizing the AEFRH epitope of the N-terminal region of Aβ_42_ [8]. In this study, we confirmed that the mRNA-based vaccine in monkeys also induces antibodies specific to the N-terminal region of Aβ_42_. Epitope mapping results revealed that while all three monkeys generated antibodies recognizing the same N-terminal region of Aβ_42_, there were slight differences in the functional role of each residue in binding with antibodies (Figure 6). For instance, antibodies from monkeys G895 and H38L recognized the epitopes AEFRHD and EFRHD, respectively, with the most crucial amino acids being EF and H. Conversely, antibodies from the G02N monkey recognized the epitope DAEFRHD, with the most crucial amino acids being D (position 1), EF, and D (position 7) (Figure 4). These findings underscore the complexity of epitope–antibody interactions and highlight the potential for individual variability in immune responses among humans.

### 3.7. Binding of Anti-Aβ Antibodies to Amyloid Plaques

**Figure 7:**
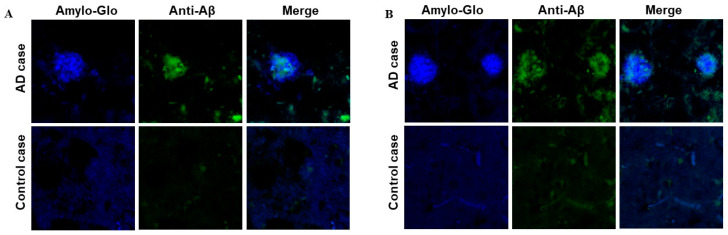
Antibodies generated by AV-1959LR (**A**) and AV-1959D (**B**) in non-human primates specifically recognize Aβ plaques in the brains of AD cases. There was no binding observed in control non-AD brain sections. Green-sera bound plaques, blue-amylo-Glo bound plaques.

Just as with mice, we assessed the binding capacity of sera from Macaca fascicularis immunized with AV-1959LR to Aβ plaques, comparing it with the sera from Rhesus macaques immunized with AV-1959D. Both sets of monkey sera effectively bound to the plaques in the brain sections from the AD case (Figure 7). We did not observe significant differences in the binding of immune sera generated by mRNA or DNA vaccines. Specifically, in this brain section, they seemed to predominantly bind to a cloud of diffuse material within the plaques.

## 4. Discussion

The era of using nucleic acids, both DNA and mRNA, as a tool for vaccination emerged as an alternative to conventional vaccines at the end of the 20th century. In 1989, Malone et al. reported on luciferase expression in mouse fibroblast cells transfected with mRNA mixed with lipofectin [25]. Importantly, in 1990, Wolfe et al. showed that mouse skeletal muscle can uptake mRNA and DNA plasmid with β-galactosidase or luciferase genes and express the protein [26,27]. Later, two groups demonstrated that in mice, DNA vaccines administrated intramuscularly induced immune responses to HIV and flu antigens, respectively [28,29]. Simultaneously, Martinon et al. demonstrated that administration of mice with a mixture of mRNA encoding flu virus nucleoprotein and liposome induced in vaccinated rodents anti-viral T cell responses [30]. Based on these data, nucleic acid immunization has received considerable scientific interest because of its significant advantages in comparison to conventional vaccines, i.e., ease of manufacturing, high stability, the capability of modifying genes encoding desired antigens, the ability to target cellular localization of an antigen by adding or removing signal sequences or transmembrane domains, and even the ability to elicit the type of immune response [31,32,33,34]. However, mRNA vaccine technology was halted for many years due to difficulties in manufacturing, the short half-life of mRNA, and its ability to activate the innate immune system. On the contrary, scientists from academia and industry have been focusing on developing naked DNA vaccines and delivery systems for this technology. As a result, several veterinary products have been licensed [35,36]. After the UPENN group reported that modifications of nucleosides of the mRNA can suppress the activation of dendritic cells [37] and increase the stability and translational capacity of mRNA [38], this vaccination strategy received a boost, leading to the emergency approval of two human vaccines worldwide for COVID-19 [39]. Similarly, the DNA vaccine delivered by the PharmaJet device system obtained emergency approval for COVID-19 in India [40,41]. 

Almost 20 years ago, in 2003, our group reported the development of the first DNA vaccine for Alzheimer’s Disease. The plasmid encoding different Aβ peptides fused with IL-4 and delivered to the abdominal skin using the Helios gene gun (Bio-Rad, Hercules, CA, USA) induced Th2-type humoral immune responses specific to the N-terminus of amyloid [42]. Subsequently, based on the AN-1792 trial data, which showed correlations between antibody titers and efficacy of amyloid clearance in vaccinated individuals, we developed a universal vaccine platform called MultiTEP for neurodegenerative disorders [6,8,10,11,21].

We developed various vaccines using this platform, including one based on a plasmid encoding three copies of the Aβ_1-11_ peptide fused with MultiTEP, AV-1959D. The efficacy and safety of this vaccine have been reported in wild-type mice, mouse models of AD, rabbits, and monkeys [6,7,8,21], and it is currently being investigated in a Phase 1 trial. In this pilot study, we generated mRNA with the same sequence, encapsulated it into LNPs, and assessed the resulting AV-1959LR vaccine’s immunogenicity in mice and monkeys.

This first mRNA vaccine, AV-1959LR, at a concentration of 5 µg, generated significantly (*p* < 0.0001) higher concentrations (119.2± 6.2 µg/mL) of antibodies in mice immunized IM compared to mice immunized IM with 10 µg naked AV-1959D delivered with a conventional syringe (18.8 ± 9.3 µg/mL) (Table 1). As expected, the delivery of AV-1959D with electroporation system TDS-IM (Ichor Medical Systems, San Diego, CA, USA) significantly increased these humoral immune responses in vaccinated mice (155 ± 22 µg/mL). Delivering 20 µg of AV-1959D with another electroporation system, BTX IM (Harvard Apparatus, Holliston, MA, USA) [43,44,45], further enhanced the immunogenicity of the DNA vaccine to a level (472 ± 229 µg/mL) [46] comparable to that in mice administered with 15 µg of AV-1959LR (678.5 ± 121.3 µg/mL). Changing the route of vaccination using the BTX ID electroporation system slightly (but not significantly) increased antibody production, with substantial variabilities in immune responses within the group (655 ± 574 µg/mL). 

Therefore, the data presented in Table 1 suggest that mice vaccinated with comparable doses of homologous DNA and mRNA vaccines generated equivalent levels of anti-Aβ antibodies. This indicates that the electroporation system is as effective in delivering double-stranded DNA plasmid into the nucleus as LNPs are in delivering mRNA nucleic acid into the cell cytoplasm of immunized mice. However, the 50 µg dose of AV-1959LR induced very high antibody production in vaccinated mice (Figure 2 and Table 1). Notably, this level of anti-Aβ antibody concentration was comparable to that detected in mice immunized with 20 µg of homologous recombinant protein vaccine, AV-1959R, formulated with Advax^CpG^ adjuvant [9,10]. 

As depicted in Figure 5 and Table 2, 100 µg of AV-1959LR administration induced high titers of IgG anti-Aβ antibodies in two out of three monkeys (average 8567 ± 5040). This suggests the activation of MultiTEP-specific CD4+ T helper cells, which we previously demonstrated with the AV-1959D vaccine [8]. The DNA vaccine delivered by the TDS IM device at doses of 400 µg and 4000 µg induced anti-Aβ antibodies in non-human primates (NHPs) with endpoint titers of 4950 ± 4264 and 7040 ± 2410, respectively (Table 2). Additionally, we assessed the immunogenicity of the DNA vaccine administered intradermally to monkeys using a high-pressure jet system (PharmaJet), which is also utilized to inject the first DNA vaccine approved in India [41]. The AV-1959D vaccine at a dose of 2000 µg induced anti-Aβ antibodies in NHPs (n = 3) with endpoint titers of 4950 ± 4264.

The clinical data on Alzheimer’s Disease (AD) immunotherapy suggest a correlation between the concentration of anti-Aβ antibodies in the periphery and the efficacy of mAb [2,24,47,48]. Our AV-1959D and the non-optimized mRNA vaccines outlined here only induce a medium level of antibodies in non-human primates (NHPs), with high variability within the group. However, our recent unpublished results demonstrate that the homologous recombinant protein AV-1959R, formulated with Advax^CpG^ adjuvant, induces high antibody titers in NHPs (endpoint titers over 32,600 ± 14,900). Nucleic acid vaccines should achieve similar antibody levels in NHPs to be effective.

The data presented in Table 1 show that a 40-times-lower dose of the mRNA vaccine AV-1959LR is as effective in generating amyloid-specific antibody responses as its DNA counterpart, AV-1959D. In mice, we demonstrated that a ~3 times dose increase of AV-1959LR enhances antibody production in vaccinated mice by 4.4 times (Figure 2 and Table 1). Therefore, one might propose that increasing the dose of the mRNA vaccine by three times could amplify antibody responses in monkeys several times. However, it is important to note that higher doses of mRNA–LNP complexes can lead to adverse effects, including activation of innate immunity, production of autoantibodies, cytokines, and chemokines, and the induction/activation of various autoimmune diseases [49]. It is important to note that the experimental mice and monkeys did not show any changes in general health status throughout the entire study period. 

## 5. Conclusions

Thus, in this pilot study, we demonstrated the feasibility of an AD vaccine based on mRNA encapsulated in LNP particles in mice and non-human primates. However, higher vaccine doses are necessary to achieve high titers of anti-Aβ antibodies. Instead of escalating the dose of this prototype mRNA–LNP vaccine, which could potentially lead to LNP toxicity, we have opted to enhance its immunogenicity by optimizing the mRNA sequence (codon usage, 3′ and 5′ UTRs, 5′-cap, polyA tail) [50,51]. This strategy aims to develop mRNA with the highest in vivo protein expression, enabling us to achieve robust humoral immune responses by injecting a minimal dose of mRNA–LNP, thus avoiding the toxicity associated with high-dose LNPs. A preventive AD vaccine developed through this strategy could stimulate long-lasting antibodies in individuals at risk of AD, potentially inhibiting the aggregation/accumulation of pathological Aβ and tau proteins and delaying the onset of dementia.

## Figures and Tables

**Table 1:** Humoral immune responses generated in C57Bl6 mice with AV-1959D DNA vaccine delivered using different routes and devices and AV-1959LR injected IM.

Nucleic Acid Based Vaccine	Mice (Immune Haplotype)	Administration			Humoral Immune Response, Binding to Aβ_42_ Peptide after 3rd Immunization		Reference
		Dose (µg/mouse)	Route	Device			
					Concentration of Anti-Aβ Antibodies, µg/mL (Average ± SEM)	Isotype (IgG1/IgG2a^b^ Ratio)	
DNA	C57BL/6 (H-2b haplotype)	20	ID	_	15.3 ± 12.2	6.3	[46]
DNA	C57BL/6 (H-2b haplotype)	20	ID	BTX	645.0 ± 574.1	8.2	[46]
DNA	C57BL/6 (H-2b haplotype)	20	IM	_	18.8 ± 9.3	1.2	[46]
DNA	C57BL/6 (H-2b haplotype)	20	IM	BTX	472.3 ± 229.6	1.1	[46]
DNA	C57BL/6 (H-2b haplotype)	10	IM	TDS-IM EP	155.1 ± 22.0	0.6	[8]
RNA	C57BL/6 (H-2b haplotype)	5 15 50	IM	LNP (ALC-0315 )	119.3 ± 6.2 678.5 ± 121.3 2998.5 ± 506.7	1.5	-

**Table 2:** Humoral immune responses generated in non-human primates with AV-1959D DNA vaccine delivered using different routes and devices and AV-1959LR injected IM.

Nucleic Acid Based Vaccine	NHP	Administration			Endpoint Titers of Anti-Aβ Antibodies after 3rd Immunization Average ± SEM	Reference
		Dose (µg/monkey)	Route	Device		
DNA	Macaca fascicularis	2000	ID	Pharmajet	4950.2 ± 4264.1	-
DNA	Rhesus macaque	400	IM	TDS-IM EP	2420.7 ± 1929.4	[7]
DNA	Rhesus macaque	4000	IM	TDS-IM EP	7040.0 ± 2410.2	[7]
RNA	Macaca fascicularis	100	IM	LNP (ALC-0315)	8567.2 ± 5040.9	-

## Data Availability

All relevant data from this study are available from the authors.
